# Nephrotic–Nephritic Syndrome Following Unilateral Nephrectomy in Wilms Tumour in a Child

**DOI:** 10.1155/crin/6427670

**Published:** 2026-04-05

**Authors:** Rawshan Zuhair Jaber

**Affiliations:** ^1^ Department of Oncology, Basrah Specialty Teaching Hospital for Children, Basrah Health Directorate, Basrah, Iraq; ^2^ Department of Pediatrics, Basrah Center for Hereditary Blood Diseases, Basrah Health Directorate, Basrah, Iraq

**Keywords:** nephrectomy, nephrotic–nephritic syndrome, Wilms tumour

## Abstract

**Background:**

Wilms tumour (WT) represents the most frequent kidney malignant tumour in the paediatric population and is occasionally linked to specific congenital syndromes. Treatment typically includes multimodal therapy consisting of chemotherapy, surgery and/or radiotherapy (RT), with a success rate of approximately 90%. The incidence of nephrotic–nephritic syndrome following unilateral nephrectomy is extremely rare; however, certain long‐term kidney complications have been documented.

**Case Report:**

A 5‐year‐old female was diagnosed with WT, Stage 3 with favourable histology. She underwent left radical nephrectomy with preoperative spillage and received RT for the whole abdomen followed by chemotherapy. She was complaint from insidious onset fever, shortness of breath, progressive generalized oedema, reddish urine and headache, occurring 5 days from the 5^th^ cycle of chemotherapy and investigation confirm diagnosis of nephrotic–nephritic syndrome.

**Conclusion:**

Kidney injury poses a risk in cases following nephrectomy, with hyperfiltration, treatment toxicity and immune effects contributing to the condition. Early detection and management are essential for kidney preservation.

## 1. Introduction

Wilms tumour (WT) is the leading malignant kidney neoplasm in the paediatric age group. Approximately 10% of the cases present with congenital anomalies, with a small proportion being a part of congenital malformation syndrome. Denys–Drash syndrome (DDS), Frasier syndrome (FS), WAGR syndrome and Beckwith–Wiedemann syndrome (BWS) are among these syndromes [1].

Currently, the treatment is successful in approximately 90% of the cases, using a combination of induction chemotherapy, nephrectomy, postoperative chemotherapy and, in certain cases, radiation therapy. The long‐term treatment‐related toxicities, particularly impairment of the remaining kidney, may adversely affect patients’ quality of life.

Nephrotoxic chemotherapeutic agents, including carboplatin and ifosfamide, are frequently used in the treatment of advanced stage or high‐risk WT and may also lead to deterioration in kidney function. RT adversely affects kidney function [2].

Nephrotic syndrome (NS) is defined by the presence of proteinuria, hypoalbuminemia, hyperlipidaemia and oedema and is the most common glomerular disease in children [1].

Nephritic syndrome, commonly referred to as glomerulonephritis, is a glomerular disorder typically manifested by oedema, hypertension and the presence of red blood cells and protein in the urine. It can be developed as a consequence of infections, inherited genetic disorders, autoimmune disorders and/or side effects of pharmaceuticals [3].

## 2. Case

A 5‐year‐old female was diagnosed with WT on 15^th^ September 2024, at Stage 3 with favourable histology. She underwent left radical nephrectomy with preoperative spillage on 24^th^ September and received RT (10.5 Gy in 7 fractions) on 14^th^ October–22^nd^ October for the whole abdomen. She commenced chemotherapy on 3^rd^ November 2024 after nephrectomy and RT.

The patient had a positive family history of kidney diseases (WT). Her sister died after relapse WT. She also had six other siblings who died with multiple congenital malformations.

The patient was admitted to the oncology department at Basrah Specialty Teaching Hospital for Children on 13^th^ January 2025 due to insidious onset fever, shortness of breath, progressive generalized oedema, reddish urine and headache, occurring five days from the 5^th^ cycle of chemotherapy (CCE: Carboplatin, Cyclophosphamide and Etoposide) as a part of UH‐1 protocol, which choice is due to previous history of unspecified congenital anomaly in the same kidney with tumour and positive family history with worst prognosis [4].

On examination, the patient was conscious and oriented but pale, experiencing generalized oedema with dyspnoea and tachypnoea. No aniridia, no hemi hyperplasia, no macroglossia, no lymphadenopathy or raised jugular venous pressure was observed. Her pulse rate was 120 bpm, respiratory rate 40 bpm, blood pressure 160/80 mmHg and oxygen saturation 90% without oxygen, which improved with oxygen supplementation. Cardiorespiratory examination revealed bilateral basal lung crepitations with a displaced apex beat downwards and pericardial rub. The abdominal examination showed symmetrical distension with ascites (positive shifting dullness and transmitted thrill) and no umbilical hernia.

Investigations included CBC, blood film, liver function tests (LFTs), kidney function tests, urine analysis, protein/creatinine ratio in urine, ANA panel, abdominal ultrasound, chest computed tomography (CT) scan with contrast and echocardiography but the gene test for WT1 could not do it (not available).

The results of the investigations are summarized in Tables 1 and 2.

**TABLE 1:** Haematological and biochemical tests.

1. CBC	On admission	On discharge
Hb (g/dL)	6.3	9.8
RBC (× 10^6^/mm^3^)	2.28	3.37
WBC (× 10^3^/mm^3^)	0.63	21.3
Neutrophil (× 10^3^/mm^3^)	0.58	19.98
Platelet (× 10^3^/mm^3^)	67	155
The results of the blood film showed pancytopenia with normocytic normochromic anaemia and no fragmented RBC or feature of haemolysis.		

**2. Liver function tests**		

AST (u/L)	22	10
ALT(u/L)	30	8
TSB(μmole/L)	13	11
S. albumin(g/L)	34	45
TSP (g/L)	57	65

**3. Renal function tests**		

B. urea (mole/L)	30	9.3
S. creatinine (mmole/L)	113	74

**4. Lipid profile**		

Triglyceride (mg/dL)	409	
Cholesterol (mmole/L)	1.6	

**5. Immunological tests**		

ANA panel	Negative	
C3 (mg/dL)	71	
C4 (mg/dL)	14	

6. Other blood test		

CRP (mg/L)	63	

*Note:* Hb; haemoglobin, AST: aspartate transaminase, ANA: antinuclear antibody.

Abbreviations: CRP, C‐reactive protein; RBC, red blood cells; TSB, total serum bilirubin; TSP, total serum protein; WBC, white blood cells.

**TABLE 2:** Significant urine analysis tests.

Urine analysis tests	On admission	On discharge
Albumin in urine	+++	Negative
RBC in urine	+++	Negative
Protein/creatinine ratio(mg/g)	12,960	−−−−−

The initial results in both tables can be summarized by pancytopenia, no features of haemolysis or fragmented red blood cells with positive C‐RP, hypoalbuminemia with hypoproteinemia, elevated renal function tests, elevated protein/creatinine ratio with albuminuria and RBC cast in urine, hypertriglyceridemia and hypocomplementemia (low C3).

Imaging studies, including abdominal ultrasound with Doppler, revealed a clear bed post left nephrectomy, with the right kidney compensatory enlarged in size and increased echogenicity, moderate to severe ascites and bilateral pleural effusion with normal ovaries and uterus and no presence of any other organ unrelated to her sex. The portal vein, kidney vessels and IVC are patent and the flow of portal vein is toward the liver.

CT of the chest (native and with IV contrast) showed pericardial and pleural effusion with pneumonia.

Echocardiography revealed mild to moderate pericardial effusion with mild left ventricular hypertrophy (LVH) and an ejection fraction (EF) of 66%.

All these results came with diagnosis of nephrotic–nephritic syndrome as early complication following unilateral nephrectomy.

Treatment commenced with dual antibiotics, albumin infusion with diuretics, oxygen supplementation, granulocyte‐colony stimulating factor, antihypertensive drugs (captopril, amlodipine, aldactone and bisoprolol fumarate), prednisolone and mycophenolate mofetil, along with blood and platelet transfusions. This treatment was administered in collaboration with nephrologists and cardiologists.

After 1 month, the patient manifested significant improvement, with controlled blood pressure, resolved oedema and pericardial effusion and continued treatment with adjusted doses according to creatinine clearance.

## 3. Discussion

The risk of end‐stage kidney disease (ESKD) is low for most patients with unilateral WT and the prevalence is quite low among those with a unilateral WT and no features suggestive of a WT predisposition syndrome (0.6%). It is higher among those with a predisposition syndrome, among those with bilateral WT and those who experience a relapse in the contralateral kidney [5].

Unilateral nephrectomy may be followed by acute kidney injury (AKI), a leading risk factor for the development and progression of chronic kidney disease (CKD), associated with multiple adverse outcomes, including cardiovascular/cerebrovascular diseases, poor health‐related quality of life, rehospitalizations and even cancer‐specific mortality, representing a growing clinical concern [6].

Evidence indicates that children who undergo nephrectomy may develop subsequent kidney complications. The reported prevalence of proteinuria, hypertension and reduced estimated glomerular filtration rate (eGFR) is approximately 15.3%, 14.5% and 11.9%, respectively. These observations emphasize the necessity for continuous long‐term surveillance of kidney function in this population [7].

The creatinine clearance (CCr) is frequently calculated using Cockcroft Gault equation, which is commonly employed as an alternative estimate of GFR in Calvert’s formula [8]. The eGFR, regardless of the equation used, is lower in survivors treated with whole abdominal radiotherapy (WART) compared with unirradiated survivors of unilateral, nonsyndromic WT [9].

Mechanisms of kidney injury following unilateral nephrectomy comprise hyperfiltration injury, secondary glomerular disease caused by increased workload on the solitary kidney that may unmask or accelerate subclinical glomerular disease, haemodynamic stress and inflammation, which can elevate glomerular capillary pressure, promote cytokine release and lead to endothelial injury, immune cell infiltration and mesangial proliferation. This may result in glomerular haematuria (nephritic) and proteinuria (nephrotic). Pre‐existing subclinical disease may become symptomatic only upon the removal of the second kidney [10].

The kidney toxicity may result from several factors, including the reduction in nephron mass following nephrectomy and exposure to nephrotoxic chemotherapeutic agents such as carboplatin, cisplatin, ifosfamide and cyclophosphamide, as well as radiation therapy when the remaining kidney lies within the irradiation field [11].

In children with WT, the likelihood of developing radiation nephropathy is considered low when the whole abdominal radiation dose is around 10.5 Gy [12]. Kidney damage secondary to RT is often accompanied by manifestations of microangiopathic haemolytic anaemia (MAHA) and thrombocytopenia. Acute radiation–related kidney injury, occurring within the first three months, is generally subclinical and associated with decline in glomerular filtration rate (GFR), and increased serum b2‐microglobulin usually develops during the subacute period (3–18 months). Chronic injury, which appears after 18 months, can include benign or malignant hypertension, elevated creatinine levels, anaemia and renal failure [13]. So, in the absence of features of MAHA, the diagnosis of radiation nephropathy was excluded.

On the other hand, the risk of atypical haemolytic uraemic (aHUS) syndrome is far away in this case because the absence of hemolysis and fragmented red blood cells (RBCs) in blood film that is an important criterion in aHUS [14, 15].

Quality of life remains a critical issue and depends mainly on adverse events and toxicity from prior treatment. Therefore, assessing the early and long‐term adverse effects is essential to improve outcomes [16].

Conventional medical treatment for nephrotic–nephritic syndrome comprises antihypertensive agents, anti‐inflammatory medications, restriction of potassium intake and rest [3].

## 4. Conclusion

This report involved a young female with WT and nephrotic–nephritic syndrome following unilateral nephrectomy. Kidney injury poses a risk in cases following nephrectomy, with hyperfiltration, treatment toxicity and immune effects contributing to the condition. Early detection and management are essential for kidney preservation. A multidisciplinary team is required.

## Author Contributions

The author contributed to planning, review and conduct of the review case report.

## Funding

No funding was received for this case.

## Ethics Statement

The ethical approval was obtained from the Committee of Iraqi Association of Medical Research and Studies (Ref. number: 1‐9/2024).

## Consent

The author has nothing to report.

## Conflicts of Interest

The author declares no conflicts of interest.
